# Pericardial mesothelioma mimicking mediastinal lymphoma and systemic rheumatic disease: a case report

**DOI:** 10.3389/fonc.2024.1481373

**Published:** 2025-01-17

**Authors:** Grzegorz Hirnle, Michał Kapałka, Michał Krawiec, Tomasz Hrapkowicz

**Affiliations:** ^1^ Department of Cardiac, Vascular and Endovascular Surgery and Transplantology, Medical University of Silesia in Katowice, Silesian Center for Heart Diseases, Zabrze, Poland; ^2^ Student Scientific Association of Adult Cardiac Surgery, Department of Cardiac, Vascular and Endovascular Surgery and Transplantology, Medical University of Silesia in Katowice, Katowice, Poland

**Keywords:** pericardial mesothelioma, constrictive pericarditis, anti-nuclear antibodies, systemic rheumatic disease, mediastinal lymphoma

## Abstract

**Introduction:**

Pericardial mesothelioma is an exceedingly rare pericardial neoplasm. It has atypical clinical symptoms and imaging characteristics that often lead to an inconclusive diagnosis. The diagnosis of a rare tumor such as pericardial mesothelioma, which can present with a variety of manifestations, requires a multidisciplinary approach.

**Case presentation:**

A 36-year-old Caucasian female patient without significant past medical history was admitted to the hospital with chest pain and a high fever and was diagnosed with acute pericarditis. The following month, the patient was treated for sepsis; during this hospitalization, lab tests for antinuclear antibodies (ANA) turned out to be positive. Concurrently, mediastinal lymphadenopathy was observed. Given the suspicion of mediastinal lymphoma, a mediastinoscopy with lymph node biopsy was performed. Following a negative biopsy result, positron emission tomography combined with computed tomography (PET/CT) and blood immunophenotyping were performed. Both tests ruled out a diagnosis of lymphoma. Concurrently, the patient was hospitalized in the rheumatology department due to positive ANA results. There, in addition to the ANA titer at a level of 1:320, lupus anticoagulant was detected. The patient was diagnosed with systemic lupus erythematosus (SLE) and initiated on chronic steroid therapy. As heart failure progressed, the patient was admitted to the cardiology department. Tissue Doppler echocardiography and cardiac magnetic resonance imaging (MRI) revealed features indicative of constrictive pericarditis. The patient underwent a pericardiectomy with satisfactory results. However, the pathology result of the pericardium remained equivocal. The patient was readmitted 3 months later with severe circulatory failure, and a salvage procedure of pericardiectomy was performed. Histopathological examination of the sections confirmed the diagnosis of pericardial epithelioid mesothelioma. The patient died after 3 weeks of palliative care.

**Conclusions:**

In the differential diagnosis of relapsing and resultant constrictive pericarditis, neoplastic processes that may mimic systemic rheumatic diseases should also be considered. Pericardial mesothelioma is a very rare diagnosis and may result in increased ANA titers, particularly anti-dense fine speckled 70 (DFS70) antibodies.

## Introduction

Constrictive pericarditis is a serious consequence of chronic pericarditis. The pericardium becomes overgrown, with fibrous thickening and also calcifications, which impairs the diastolic function of the heart. Rarely, the cause of constrictive pericarditis may be neoplastic processes. Primary cardiac neoplasms are rare entities with a prevalence of 0.001%–0.056% and account for 0.3% to 0.7% of all cardiac cancers (1). Pericardial mesothelioma has atypical clinical symptoms and imaging characteristics that often lead to inconclusive diagnosis. The lack of effective chemotherapy treatment is associated with a poor prognosis (2). This case study presents a detailed diagnostic pathway of a patient with pericardial mesothelioma, which manifested as lymphoma and rheumatic disease.

## Case presentation

**Figure 1:**
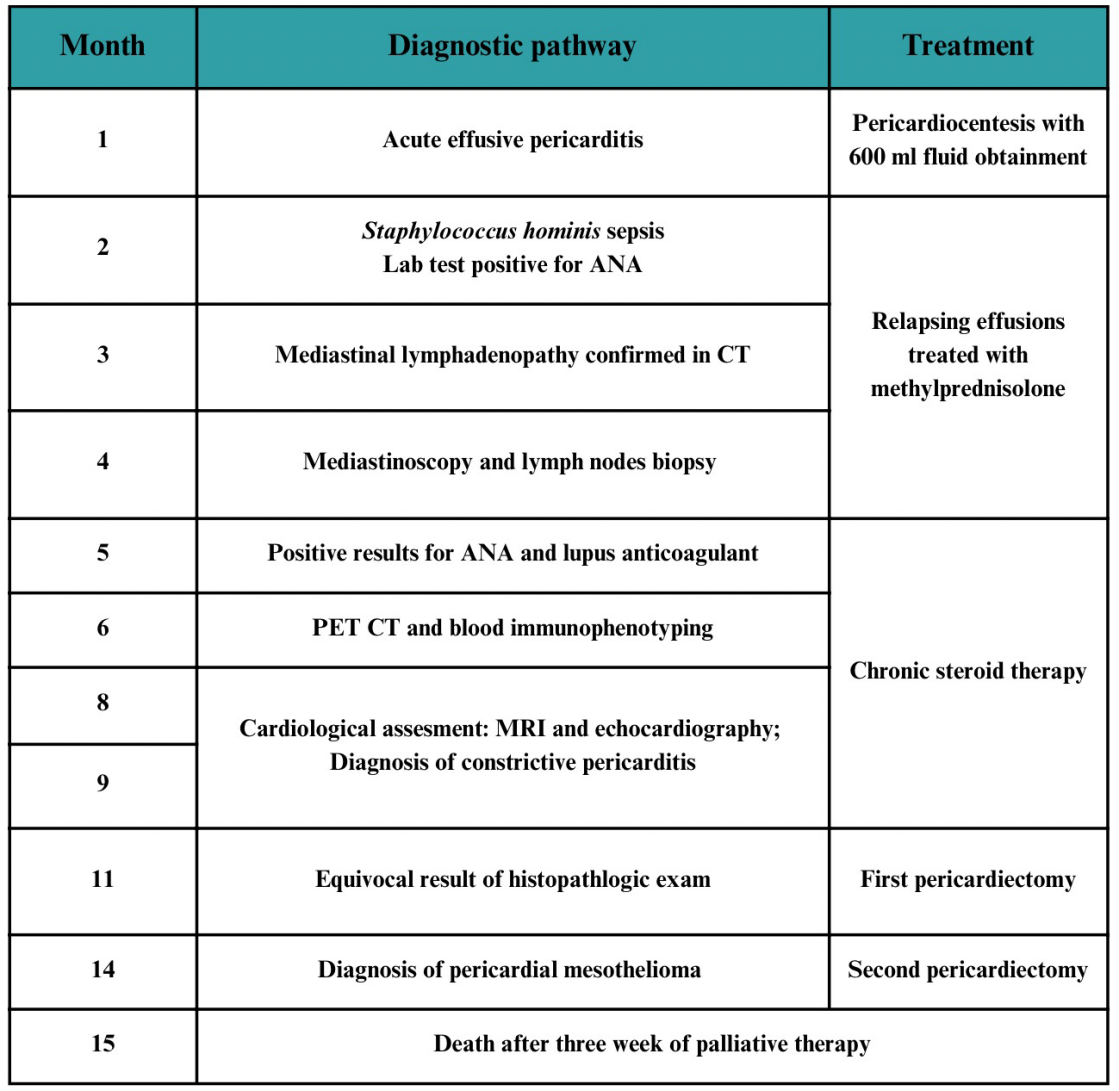
Timeline presenting the diagnostic and therapeutic process.

A 36-year-old female patient with a history of bilateral conjunctivitis (8 months earlier) was admitted to the hospital with chest pain and high fever. She was diagnosed with acute pericarditis. Pericardiocentesis was performed, yielding 600 mL of fibrinous exudate, with negative microbiological results (**Figure 1**).

One month later, the patient was readmitted with symptoms of a very severe pulsatile headache, which did not respond to standard pain treatment. Neuroinfection was ruled out by the cerebrospinal fluid examination and imaging studies. Based on elevated inflammatory markers and one positive blood culture for *Staphylococcus hominis*, antibiotic therapy with cloxacillin was initiated. The patient was treated for 14 days for bacteremia of unknown origin.

Additional investigations were performed at this time. In echocardiographic assessment, no vegetations were seen on cardiac valves, and the pericardium was slightly thickened with trace amounts of fluid (2 mm). Laboratory tests revealed antinuclear antibodies (ANA) with a nuclear dense fine speckled type. A computed tomography (CT) scan showed mediastinal lymphadenopathy with lymph nodes up to 2 cm.

During a subsequent hospitalization due to severe dyspnea, in the following month, CT scans confirmed mediastinal lymphadenopathy, characterized by multiple lymph nodes measuring 1–2 cm in size, as well as a new 85 × 56 mm tumor not seen on a previous CT scan, located anterior to the ascending aorta. Complex lab tests were performed (**Table 1**). Because of the suspicion of mediastinal lymphoma, mediastinoscopy with lymph node sampling was performed. Histopathological analysis showed only reactive lesions. Immunohistochemical examination revealed expression of the following molecules: CD20 (+), CD30 (−), CD15 (−), CD3 (−), BCl-2 (+), and Ki 67 (+).

**Table 1:** Results of the lab tests performed.

Parameters	Results	Reference values
Selected lab tests performed in connection with suspected lymphoma		
IgA	4.78 g/L	0.65–4.21 g/L
IgG	13.4 g/L	5.52–16.31 g/L
IgM	2.14 g/L	0.33–2.93 g/L
Free Kappa chains	28.3 mg/L	3.3–19.4 mg/L
Lactate dehydrogenase	284 U/L	<284 U/L
ANA	1:320 dense fine speckled (DFS) pattern	<1:80
Blood immunofixation	Negative for monoclonal proteins	
Lab tests performed at the rheumatology department		
CRP	12 mg/L	<5 mg/L
Erythrocyte sedimentation rate	61 mm/h	3–15 mm/h
NT-pro-BNP	388.8 pg/mL	<125 pg/mL
β-2-microglobulin	2.21 mg/L	1.09–2.53 mg/L
β-2-glycoprotein IgG	22.4 U/mL	<20 U/mL
β-2-glycoprotein IgM	4.9 U/mL	<20 U/mL
ANA	1:320 DFS pattern	<1:80
ANA immunoblot antigens	Dense fine speckles +++	–
Anti-CCP antibodies	<0.5 U/mL	<5 U/mL
pANCA antibodies	4.11 RU/mL	<20 RU/mL
cANCA antibodies	2 RU/mL	<20 RU/mL
Rheumatoid factor	7.9 IU/mL	<14 IU/mL
Lupus anticoagulant (ratio)	1.67	0–1.2
Anticardiolipin antibodies IgG	26.4 U/mL	<20 U/mL
Anticardiolipin antibodies IgM	7.8 U/mL	<20 U/mL
Complement C4	0.26 g/L	0.15–0.57
Complement C3c	1.21g/L	0.83–1.93

The following month, positron emission tomography with computed tomography (PET/CT) scan was performed to exclude lymphoma, which ruled out this diagnosis. The tumor located anteriorly to the aorta described in a previous CT scan turned out to be a normodense fluid reservoir with reduced dimensions of 44 × 35 mm with reduced fluorodeoxyglucose uptake. However, the maximal standardized uptake value (SUVmax) in the pericardial sac was 8.7, which was interpreted as an inflammatory lesion. The final exclusion of lymphoma was made based on the performed blood immunophenotyping.

During a 3-month period of hematological diagnostic evaluation, the patient was concurrently hospitalized in the rheumatology department. The patient still experienced fever episodes and relapsing pericardial effusions treated *pro tempore* with methylprednisolone. There, in addition to the ANA, lupus anticoagulant was detected and a diagnosis of systemic lupus erythematous (SLE) was made based on the EULAR criteria (3). In view of the patient’s abnormal Schirmer test, Sjögren’s syndrome was also suspected. Since that time, the patient had been on chronic treatment with prednisone. Interestingly, the patient exhibited laboratory features of antiphospholipid syndrome, with positive results for β-2-microglobulin, β-2-glycoprotein, and lupus anticoagulant; however, these features did not persist for more than 12 weeks. Additionally, the patient showed no clinical features of the syndrome. Throughout the aforementioned period of hematology and rheumatology evaluation, the patient had persistently elevated C-reactive protein (CRP) levels, and the ANA titer increased to the 1:3,200 level.

**Figure 2:**
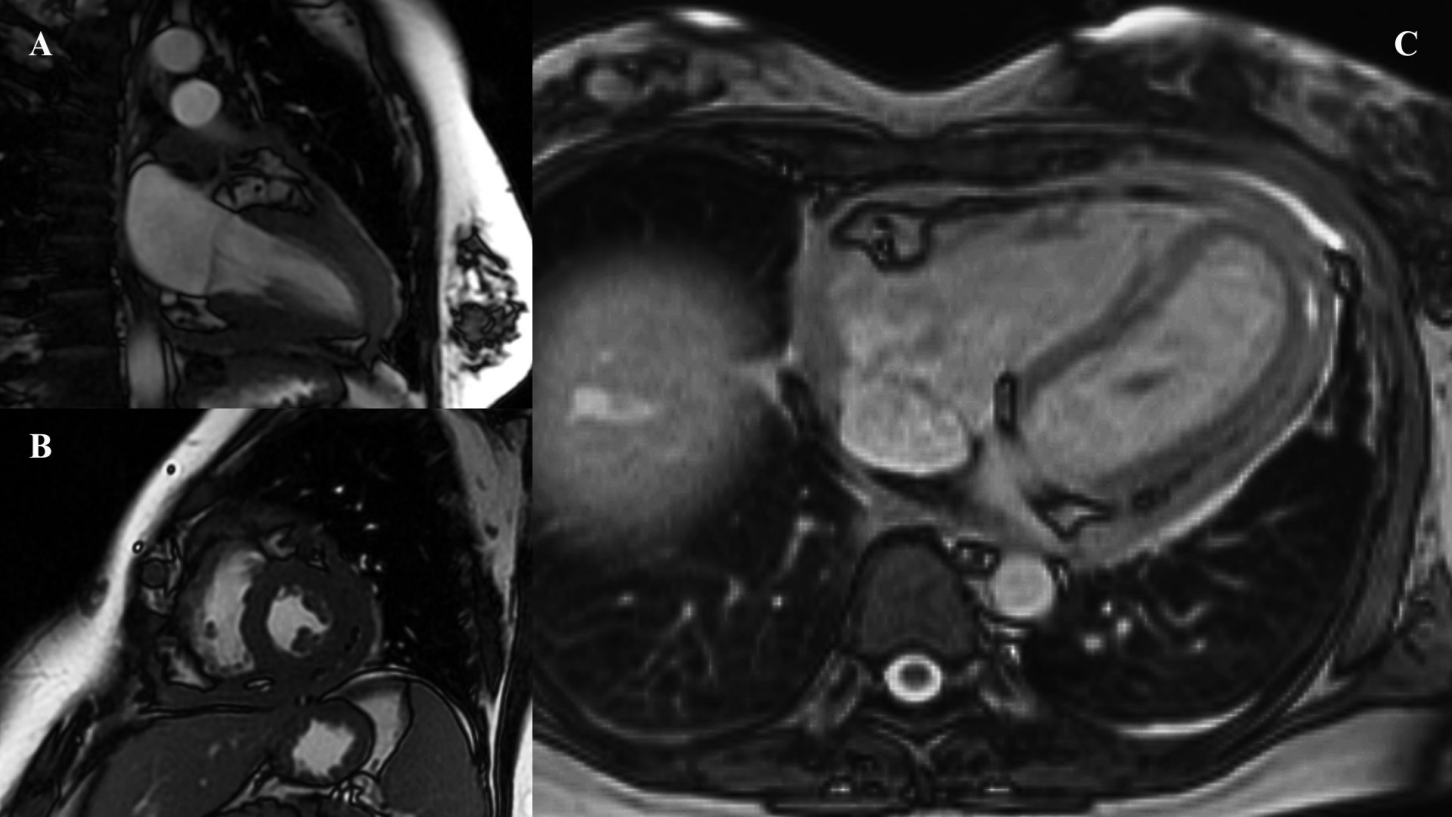
**(A, B)** First performed MRI scan—short and long axis. **(C)** MRI performed before pericardiectomy.

At 6 months, as heart failure progressed, the patient was admitted to the cardiology department for further treatment. Strain echocardiography, tissue Doppler echocardiography, and cardiac magnetic resonance imaging (MRI) revealed features indicative of constrictive pericarditis. MRI showed a hyper-intense space around the entire heart with separation up to 9 mm in front of the right atrium (RA), up to 9 mm on the diaphragmatic wall of the right ventricle (RV), and up to 8 mm behind the posterolateral wall of the left ventricle (LV) (**Figure 2**). Numerous hypo-intense bands were noted, suggesting dense, organized fluid with numerous fibrous bands. The visceral pericardium was thickened up to 5 mm around the entire heart. After intravenous administration of a contrast agent, there was marked enhancement of the pericardial lamina around the whole heart. The patient was accepted for pericardiectomy.

It was decided to re-evaluate the diagnosis of SLE. The patient denied skin rashes, psoriatic lesions, Raynaud’s sign, muscle weakness, sacral pain, or morning stiffness. At that time, the patient also no longer met the criteria for the active disease process of SLE in the SLE Risk Probability Index. The patient was evaluated for 4.5 points: 1.5 points for serositis and 3 points for ANA. A score >7 points is indicative of a diagnosis of SLE. Sjögren’s syndrome was also excluded as anti-Ro and anti-La antibodies remained negative.

The pericardial sac was excised from the anterior wall, and the inferior and superior vena cava outlets were dissected, including the pulmonary artery. Postoperative period was uneventful and an improvement in diastolic cardiac function was achieved. Histopathological analysis of the excised material remained inconclusive. Inflammatory–necrotic lesions were observed, along with resorptive granuloma featuring a purulent inflammatory infiltrate. Cell atypia of a very minor degree, with a reactionary character, was noted with low mitotic index (one mitotic figure per 10 high-power fields). Results of immunohistochemistry showed positive Calretinin, WT1, and BAP1 with negative BerEP4, classifying the immunoprofile as equivocal. No membrane staining was observed in epithelial cells during the PD-L1 22C3 qualitative immunohistochemical assay.

However, the patient was readmitted 3 months later with severe circulatory failure. After obtaining the patient’s consent, a repeat pericardiectomy was performed as an immediate salvage procedure. Perioperative risk in EuroSCORE II (European System for Cardiac Operative Risk Evaluation) reached 28%. The procedure revealed massive adhesions from the previous surgery and thick calcified pericardial laminae fused together with the myocardium. During the reoperation, sections were taken for histopathological examination, the result of which revealed the diagnosis of pericardial epithelioid mesothelioma. It is worth mentioning that the patient did not report a history of asbestos exposure.

**Figure 3:**
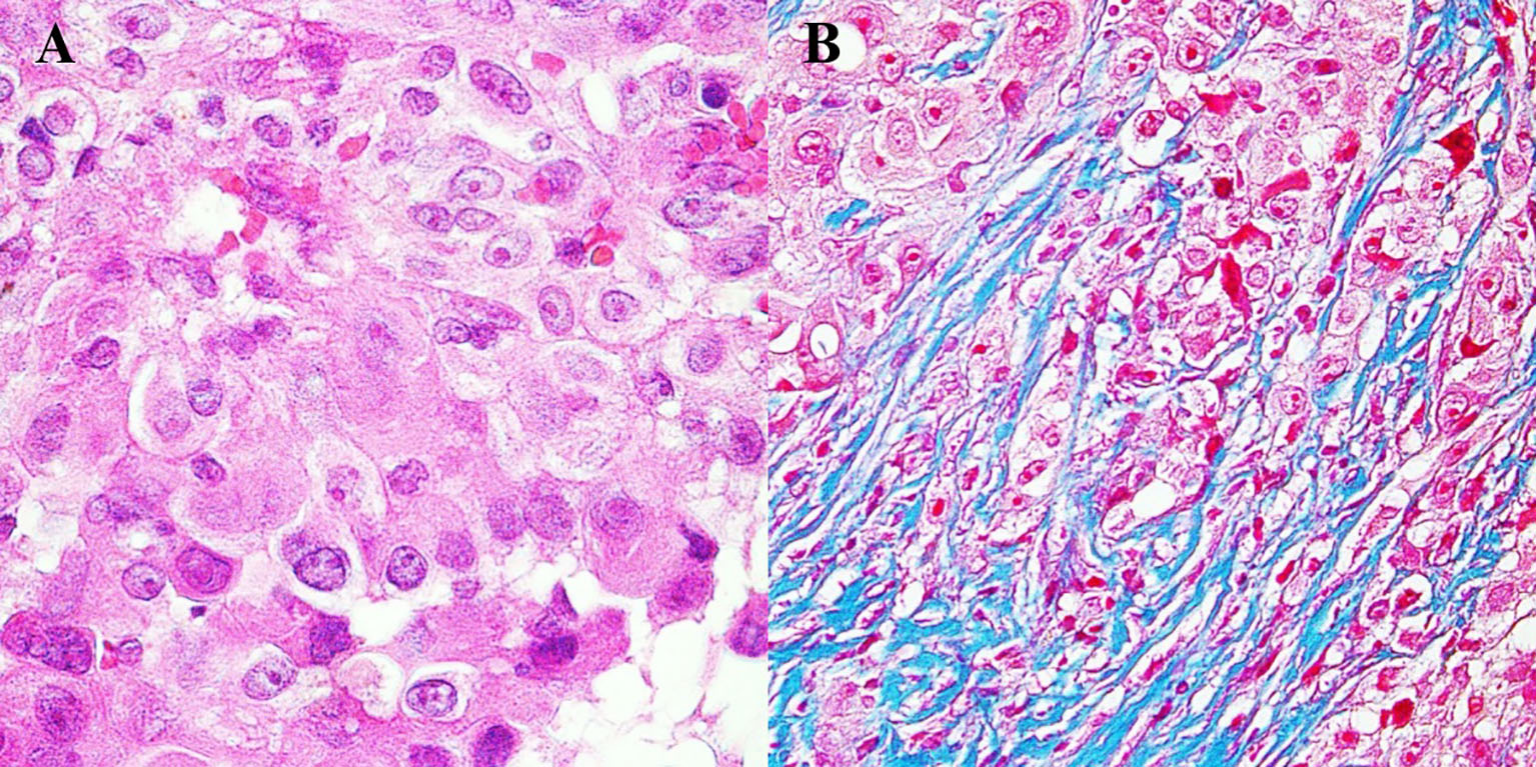
Histopathological image of mesothelioma. **(A)** Tumor cells with visible nuclei. Hematoxylin and eosin staining, magnification 500×. **(B)** Tumor cells infiltrating connective tissue fibers. Masson staining, magnification 500×.

Pathomorphological exam revealed neoplastic cells with marked atypia (**Figure 3**). Immunohistochemistry reaction showed positive results for the following: BAP1, Calretinin, CK 5/6, Podop (D-20), WT1 (+), and Ki-67 at the level of 20%.

The patient was disqualified from oncological treatment due to the lack of effective chemotherapy and the fact that pericardiectomy is the treatment of choice. The patient died after 3 weeks of palliative care.

The timeline of the diagnostic and therapeutic pathway is presented in **Figure 1**.

## Discussion

Primary pericardial mesothelioma (PPM) originates from the mesothelial cells of the pericardium. It constitutes less than 1% of all malignant mesotheliomas but accounts for nearly 50% of all primary pericardial tumors (4). Risk factors for pericardial mesothelioma are debated, although the most commonly reported are asbestos exposure, radiotherapy (including breast cancer treatment), chemotherapy, smoking, and a history of cardiovascular diseases (5), but the patient described had no such history.

During the diagnostic process of the patient, at the time of relapsed pericarditis, presumed diagnoses of mediastinal lymphoma and SLE were made before the eventual diagnosis of pericardial mesothelioma was established. Negative PET/CT was interpreted for exclusion of lymphoma, and due to positive ANA, SLE remains as an alternative diagnosis. At that time, engaging a multidisciplinary team discussion could have provided valuable insights, potentially suggesting the necessity of multimodal imaging or facilitating a more thorough evaluation of the likelihood of an alternative diagnosis of SLE.

### Lymphoma as the initial diagnosis

Initially, the clinical presentation raised strong suspicion of mediastinal lymphoma, with features such as mediastinal lymphadenopathy with pericardial involvement. Mediastinal lymphadenopathy may indicate a malignant process, such as mediastinal lymphoma. Notably, secondary cardiac lymphoma occurs in approximately 9%–24% of patients with lymphoma (6). The pericardium is the most commonly involved cardiac structure, often leading to pericardial effusion (7) and, in severe cases, cardiac tamponade (8) (9). Interestingly, pericardial involvement has also been reported as the initial presentation of lymphoma in some cases, underscoring its diagnostic significance (10). Given that secondary pericardial involvement is far more common than primary pericardial malignancies, a hematologic diagnostic pathway remains a rational and essential approach for the described patient. Further investigation focused on exclusion of the more common diagnosis through biopsy, PET/CT, and blood immunophenotyping.

### Suspicion of SLE

The case described above highlights that other cancers can also arise in the presence of positive ANA. Several cases have reported systemic lupus erythematosus (SLE)-like features associated with pericardial mesothelioma. McGuigan described a case of PPM presenting with SLE-like symptoms but with low titers of ANA (11). Similarly, Mensi reported a case of PPM characterized by non-erosive polyarthritis, photosensitive rash, sicca syndrome, and recurrent episodes of pericarditis with pericardial effusion. This patient also exhibited high ANA titers, similar to the case described earlier (12). Additionally, Rakhra documented a case of pleural mesothelioma presenting with SLE seropositivity, where serological findings included positive ANA, low-titer anti-double-stranded DNA antibodies (15 IU/mL), and rheumatoid factor (RF) (16 IU/mL) (13). In contrast, the patient in our case had positive ANA, negative RF, and positive lupus anticoagulant. The diagnostic challenge posed by these overlapping features, along with the risk of misdiagnosis, often leads to the late identification of the cancer, contributing to a high mortality rate.

ANA may be associated with a variety of cancers and may have potential anti-tumor activity on an antibody-dependent cell-mediated cytotoxicity (14). The development of autoantibodies results from the breakdown of immunological tolerance, stemming from B- and T-cell dysregulation (15, 16).

The study by Solans-Laqué showed ANA seropositivity with a prevalence of 43.7% in gynecological cancers and 26.6% in lung cancer (17). The presence of ANA is particularly common in patients with lymphomas, with reports indicating a prevalence as high as 31.5% (18). Barreno-Rocha reported the presence of both lupus anticoagulant and ANA in 23.3% of the analyzed patients (19). In the patient with coexisting mediastinal lymphadenopathy, this condition initially led clinicians to suspect lymphoma. Since lymphoma can metastasize from the mediastinum to the pericardium, it should be excluded through biopsy, immunophenotyping, and PET.

Determining the specific type of ANA against nuclear antigens is also crucial in the further rheumatologic diagnostic process. The patient described above had a homogeneous and speckled luminescence pattern of ANA. Notably, in Gauderon’s analysis, the presence of this particular pattern was significantly associated with the absence of cancer, a finding that was not confirmed by the patient’s clinical course (20). Conversely, Cheng’s meta-analysis identified anti-dense fine speckled 70 (DFS70) antibodies as having high specificity for excluding systemic autoimmune rheumatic diseases (21). However, it is important to note that positive DFS70 antibodies can occur in 3.2% of SLE cases and in 10% of Sjögren’s syndrome cases (22). Therefore, their presence cannot unequivocally rule out the possibility of diagnosing these diseases.

The anti-DFS70 antibody, also known as DFS70, Lens epithelium-derived growth factor (LEDGF), or DNA-binding transcription co-activator p75, is an autoantibody closely associated with the dense fine speckled (DFS) pattern (23). DFS70 is overexpressed in various cancers and has oncogenic functions as an oncoprotein, participating in the transcriptional activation of cancer-associated genes and mRNA splicing (24). It promotes cancer cell proliferation and enhances the tumorigenic and metastatic properties of neoplasms (25).

### Diagnosis and difficulty in diagnosis of pericardial mesothelioma

Constrictive pericarditis is a typical manifestation of pericardial mesothelioma (2). It is a complication of chronic or recurrent pericarditis; thus, it is likely to be a symptom that appears at a late stage of cancer development. Symptoms of tamponade may also be present, sometimes being the first manifestation of the disease (26). Analyzing the case retrospectively, early signs of mesothelioma included a large pericardial effusion, noticeable pericardial thickening, and mediastinal lymphadenopathy, observed alongside the exclusion of more common causes, such as metastasis from lymphoma.

Unfortunately, the usefulness of both biopsy and pericardial fluid cytology is limited. The diagnostic yield of pericardial fluid cytology is often low, with only 24% of cases showing malignant cells, according to Nilsson’s analysis (27). The false-negative rates of pericardial biopsy in detecting malignant pericardial effusions have been reported to be 40%–44.7% (28). In cases of constrictive pericarditis, the pericardium typically exhibits thickening due to collagen fibrosis with areas of hyalinization, thick-walled blood vessels, and minimal chronic inflammation (29). As highlighted in the case report, such histological findings can introduce significant challenges in the evaluation of sections taken during pericardiectomy. This can delay the diagnosis, which ideally should be made before pericardial constriction occurs, as it complicates the ability to perform radical resection of neoplastic tissue. Tissue samples may be taken from fibrotic areas without obvious atypia, underscoring the need for very precise and extensive sampling during the pericardiectomy procedure.

Other considerations should be given to the immunohistochemistry of mesothelioma lineage. What makes the case described interesting is the equivocal immunoprofile of the pericardial tissue taken during the first pericardiectomy. Such results caused a delay in the diagnosis and treatment of the patient. The negligible atypia suggested inflammatory changes in the mesothelium. Benign reactive mesothelial proliferation, even with atypia, may be caused by infection, collagen vascular disease, surgery, or trauma (30). The tissue showed low mitotic activity—one mitotic figure per 10 high-power fields, compared to other pericardial mesothelioma cases reported by Karadžić, which had five mitotic figures per 10 high-power fields (31). Elliot asserts that mitotic activity may be increased in mesothelial hyperplasia; however, atypical mitoses should not be present (32). In such ambiguous situations, an immunohistochemical reaction is indicated. A positive reaction for WT1 and Calretinin confirms mesothelial proliferation, while the absence of BerEP4 indicates no epithelial proliferation (33). The loss of BAP1 is known as a highly specific marker for distinguishing malignant mesothelioma from reactive proliferation (34). At that time, considering the rarity of mesothelioma, the patient’s tissue was classified as inflammatory mesothelial proliferation.

In patients with recurrent pericardial effusions of unclear etiology, multimodal imaging plays a pivotal role in establishing a definitive diagnosis. Among these modalities, cardiac MRI is particularly valuable in the assessment of pericardial tumors, offering superior anatomic delineation and the ability to characterize tissue composition. It also aids in identifying tumor infiltration, as well as necrotic and fibrotic lesions (35, 36).

Pericardial mesothelioma, a rare and aggressive tumor, typically appears homogeneously isointense on T1-weighted images and heterogeneous on T2-weighted images, with gadolinium enhancement that can sometimes be irregular due to necrotic tissue components (36–38). These imaging features are critical in distinguishing mesothelioma from other pericardial pathologies.

Liu et al. emphasized the importance of multimodal imaging in such cases, recommending the combined use of echocardiography, contrast-enhanced echocardiography, cardiac MRI, and PET/CT to achieve a comprehensive diagnostic evaluation (36). This approach not only enhances diagnostic accuracy but also helps to determine the extent of local invasion and the presence of distant metastases, which are key to planning appropriate management strategies.

The role of PET/CT in diagnosing mesothelioma warrants attention. In the current case, the patient’s thickened pericardium, measuring 18 mm and exhibiting an SUVmax of 8.7, was interpreted as indicative of chronic pericarditis. However, reported cases utilizing PET/CT for mesothelioma diagnosis have typically shown an SUVmax greater than 10. Notably, some studies have documented increased diffuse uptake in the pericardium, with SUVmax values reaching 19.5 in both the right and LVs (39), as well as a localized pericardial mass with an SUVmax of 12.9 (40). In contrast, Hyeon’s analysis revealed that among 11 patients with malignancy, the median SUVmax was only 3.4, suggesting that the interpretation of the current patient’s results may not be accurate (41).

The primary treatment is excision of the tumor through pericardiectomy; however, this approach is beneficial only for localized tumors and before the potential process of constriction begins (42). There are also reported cases of response and prolonged survival in patients treated with pemetrexed and platinum-based chemotherapy (43, 44).

## Conclusions

Following recurrent effusive or constrictive pericarditis with no convincing etiology diagnosis, unexplained laboratory or imaging abnormalities should prompt a review by a multidisciplinary team to guide further diagnostic pathways. Multimodality imaging, including PET/CT, MRI, and echocardiography, is essential to consider rare diagnoses such as pericardial mesothelioma.

## Data Availability

The raw data supporting the conclusions of this article will be made available by the authors, without undue reservation.
